# The Common Cichory (*Cichorium intybus* L.) as a Source of Extracts with Health-Promoting Properties—A Review

**DOI:** 10.3390/molecules26061814

**Published:** 2021-03-23

**Authors:** Katarzyna Janda, Izabela Gutowska, Małgorzata Geszke-Moritz, Karolina Jakubczyk

**Affiliations:** 1 Department of Human Nutrition and Metabolomics, Pomeranian Medical University in Szczecin, 71-460 Szczecin, Poland; Katarzyna.Janda@pum.edu.pl (K.J.); jakubczyk.kar@gmail.com (K.J.); 2 Department of Medical Chemistry, Pomeranian Medical University in Szczecin, 70-111 Szczecin, Poland; 3 Department of Pharmacognosy and Natural Remedies, Pomeranian Medical University in Szczecin, 70-111 Szczecin, Poland; mgeszke@pum.edu.pl

**Keywords:** chemical composition, characteristics, antiviral properties, SARS-CoV-2, application

## Abstract

Natural products are gaining more interest recently, much of which focuses on those derived from medicinal plants. The common chicory (*Cichorium intybus* L.), of the Astraceae family, is a prime example of this trend. It has been proven to be a feasible source of biologically relevant elements (K, Fe, Ca), vitamins (A, B_1_, B_2_, C) as well as bioactive compounds (inulin, sesquiterpene lactones, coumarin derivatives, cichoric acid, phenolic acids), which exert potent pro-health effects on the human organism. It displays choleretic and digestion-promoting, as well as appetite-increasing, anti-inflammatory and antibacterial action, all owing to its varied phytochemical composition. Hence, chicory is used most often to treat gastrointestinal disorders. Chicory was among the plants with potential against SARS-CoV-2, too. To this and other ends, roots, herb, flowers and leaves are used. Apart from its phytochemical applications, chicory is also used in gastronomy as a coffee substitute, food or drink additive. The aim of this paper is to present, in the light of the recent literature, the chemical composition and properties of chicory.

## 1. Introduction

The name of the common chicory plant (*Cichorium intybus* L.) most probably derives from several Greek and Latin words. *Cichorium* is Latin for “field”, while *intybus* finds a counterpart in both languages. The Greek counterpart means “to cut”, and pertains to the morphology of the leaves. In turn, the Latin *tubus*—meaning “(a) tube”—describes the structure of the stem [1]. The name of the genus is representative of its habitat, while the species name describes the plant itself [1]. The name *Cichorium* was in fact created by latinization of the Greek *kichorion*, oftentimes encountered in works by ancient medics. It is an archeophyte of Mediterraneo-irano-turan origin. The plant boasts a long history of almost universal application, though its medical use is particularly interesting, as it dates back to prehistorical times. In ancient Rome, Greece and Egypt it was used for boosting metabolism and digestion. The plant was used as a vegetable as well as a pasture plant [1,2]. Many accounts exist of its medical properties, i.a. from Theophrastus of Eresos, Pedanios Dioscorides, Pliny the Elder or Avicenna, who used chicory preparations for treatment of digestive or visual disorders. Avicenna also applied chicory root decoctions for alleviation of gout pain. (*Cichorium intybus*) has been extensively used for a long period in the Unani system of medicine, too [3]. Apart from the abovementioned, medieval Europeans would use chicory to treat jaundice, or even malaria [4,5,6]. Cultivated chicory can be sorted into groups, according to their use: (1) “Industrial” or “root” chicory, grown mostly in northwestern Europe, India, South Africa and Chile; it is mostly used for extraction of inulin or production of a coffee substitute from the plant’s taproot; (2) “Brussels” or “witloof” chicory, the most common industrial European variety; its buds are bleached by storing in a sun-deprived place; (3) “Leaf” chicory, used fresh or boiled; (4) “Pasture” chicory, derived from the wild chicory, common along roads and wastelands [1,5,7]. Commonly it is known as chicory, blue sailors, succory, coffee weed, cornflower, wild chicory, wild succory, garden endive, garden chicory, endive, blue daisy, blue dandelion, blue weed, bunk, hendibeh, horseweed, ragged sailors, blue sailors, wild bachelor’s buttons, witloof and wild endive [7,8,9]. The occurrence of this species was documented in Northern and Central Europe, Siberia, Turkey, Afghanistan, North and Central China, South America, South Africa, Ethiopia, Madagascar, India, Australia, New Zealand [7]. *Cichorium intybus* L. is thus an important medical plant in all of Eurasia and some of Africa. Despite a long tradition of its application, however, it is not included in the European Pharmacopoeia, nor in any of the pharmacopoeias of the EU member states. It is however widely used in traditional medicine thanks to the widespread trade of its particular parts [1,8].

## 2. Botanical Characteristics

*Cichorium intybus* L. is an annual, biennial or perennial plant belonging to the family *Asteraceae* (formerly: *Compositae*) [9]. It is winter hardy and usually reaches 20 to 150 cm in height. It forms a long and strong, spindly, thick, brown tapio root. The stem and vein are usually green, although they can take on an occasional red ring. *Cichorium intybus* can grow from many tall, empty, ribbed stems, each of which can be lifted or rising. It is stiff and has few leaves. At the bottom of the stem there are short, thick hairs, and at the top there are most branches. The buds are branched, stiff and contain no juice. The leaves of *C. intybus* are green, arranged in a 10–25 cm long rosette. Their shape is narrowly oval, oblong, lanceolate, usually pinnate or serrated. The leaf hair may be present on the lower side of the leaf, mainly on the nerve, on the whole leaf surface or be absent at all. The lower leaves are caudal, with a very wide top segment, while the side segments are triangular, with the apex facing the base of the leaf. The leaves of the stem are green with gray shades, oblong, lanceolate, with an arrow or heart shaped base. The edges are serrated, with teeth decreasing towards the top of the leaf. The upper leaves are whole on the edges and on the surface ciliate. The inflorescence of *C. intybus* L. consists of numerous, multi-flower anthodiums 2–4 cm in diameter, set on short, thick peduncles either singly or gathered in groups of several people. The color of the marginal flowers is light blue, while the central tube flowers are darker. The flowers open only on sunny days. They are particularly rich in nectar and pollen. The cichory plant gives a light brown shell 2–3 mm long, triangular or pentagonal with short pappus hairs. The stalk is topped with a circle of scales in the upper part. *C. intybus* grows wild across the temperate climate zone of Europe, southwest Asia, in the Ural and in north Africa. It has also spread to Australia, New Zealand and the Americas. In a temperate climate zone it is common not only as a wild plant, but also as a cultivated variety. It inhabits meadows, wastelands, fallows, grasslands and trackways, both in lowlands and lower mountain regions [3,7,8,9,10].

## 3. Chemical Composition

All morphological parts of chicory (roots, herb, flowers and leaves) contain a large number of various chemical compounds. Cichoric acid was found to be the main component of the methanolic extract from *C. intybus* L. Aliphatic compounds and their derivatives are the main fraction, with terpenoids slightly less abundant in the plant. Roots of chicory contain, amongst others, sap, sesquiterpene lactones, such as e.g., germacranolides (lactucin, lactucopicrin and 8-deoxylactucin) as well as guajanolides (cycriozides B and C, sonchuzide C), which gives a bitter taste. Also, taraxane-type triterpenes can be found here, such as taraxasterol, phenolic acids (e.g., chlorogenic, isochlorogenic, neochlorogenic, caffeic and cichoric acids) The root contains 0.01–0.02% of the bitter intybin glycoside (whereby the herb contains between 0.1–2.0% of this chemical), 9–15% reducing sugars, and between 40–60% of inulin (as the plant energy store); however, no starch is present in chicory roots. Also worth mentioning are: intybinene, a common component of the coffee substitutes, pectins, vitamins B and C [2,11]. Chicory leaves contain inulin, A, B_1_, B_2_ and C vitamins, Ca, K, Mg, Na, Fe, Cu, Mn, Zn, phenolic compounds, amongst others [11].

Flowers contain various sugars, coumarin derivatives (e.g., umbelliferone, esculin, cicorin (esculetin 7-O-glucoside, scopoletin), silicic acid, taraxosterol, valeric acid, flavonoids (hyperoside), etheric oils and anthocyanins, the latter of which bring about the perianth’s blue color [12]. Also present in the plant are: gum, choline, phytosterols, mucus, tannins, copper, latex, lipids, proteins, P and K vitamins, amino acids, β-sitosterol, malic acid, oxalic acid, shikimic acid, quinic acid, succinic acid, tannins, saponins, flavonoids, terpenoids, cardiac glycosides, anthocyanins [2,7,8,9,11,13,14,15,16,17]. Selected chemical components found in chicory are presented in Table 1.

The aerial parts and roots are also a source of essential oils, too [1,26,27]. The essential oils isolated from *Cichorium intybus* are shown in Table 2. These compounds are present in various amounts, which depend on both the part of the plant and the origin of the chicory

Essential oils have a large spectrum of activity, especially antiparasitic and antimicrobial [29]. In the available literature, no results were found on the study of the properties of essential oils isolated from chicory.

## 4. Health-Promoting Properties

Fresh and dried material is the most commonly used for medicinal purposes [1,30]. It is one most important therapeutic plant which belongs to family Asteraceae [15]. Various *C. intybus* extracts have demonstrated a wide range of biological and pharmacological properties viz., anti-hyperuricemia, antiinflammatory, and antidiabetic, antinematodal, antioxidant and antiproliferative, hepatoprotective antibacterial antiprotozoal effects [1,8,10,31,32,33,34]. The plant also shows laxative, detoxifying, invigorating, blood-cleansing and anti-oxidative properties. Also observed is the diuretic effect, attributed to phenolic acids. In the Unani and Ayurvedic medicine systems the seeds of *Cichorium intybus* have been used efficaciously [15]. High levels of inulin facilitate the development and maintenance of proper gut microbiota, thus giving the plant a “probiotic” label [2,35,36].

### 4.1. Antiviral Properties

The extracts of *Cichorium intybus* L. showed great anti viral activity against HSV-1 and partial activity against adenovirus at higher concentrations [37]. In the study Zhang et al., the anti-hepatitis B property of cichoric acid was evaluated by the D-galactosamine (D-GalN)-induced normal human HL-7702 hepatocyte injury model, the duck hepatitis B virus (DHBV)-infected duck fetal hepatocytes and the HBV-transfected cell line HepG2.2.15 cells, respectively. This study verifies the anti-hepatitis B effect of cichoric acid from *C. intybus* leaves, therefore cichoric acid could be used to design the antiviral agents [38]. 

Since the outbreak of the coronavirus disease (COVID-19) caused by SARS-CoV-2 in December 2019, there has been no vaccine or specific antiviral medication for treatment of the infection where supportive care and prevention of complications is the current management strategy. Shawky et al. [39] using a molecular docking method investigated the potential of using medicinal plants in alleviating the novel SARS-CoV-2 infection. A database comprising more than 16,000 compounds was compiled and docked against the crucial viral proteins; 3-chymotypsin protease (3CLpro), papain-like protease (PLpro) and RNA-dependent RNA polymerase (RdRp) to find potential inhibitors for these enzymes. Furthermore, network pharmacology analysis of all the compounds in the database was attempted to speculate those that can target the inflammatory and immunity-related pathways at the molecular level and revealing those plants with potential multi-components and multi-targets that may help regulate the body functions and possibly will play a therapeutic role in the mitigation of the disease. *C. intybus* was found, along with *Glycyrrhiza glabra* and *Hibiscus sabdariffa* among plants with high antiviral potential. Chicory is a rich source of caffeic acid with antiviral properties. The authors suggest that further in vitro and in vivo analyses are needed to confirm the findings. Nevertheless, chicory was among the plants with potential against SARS-CoV-2 [39,40,41]

### 4.2. Antibacterial and Antifungal Properties

Chicory plant was active against gram positive and negative bacteria and yeast. C. intybus extracts inhibits Agrobacterium radiobacter sp. tumefaciens, Erwinia carotovora, Pseudomonas fluorescens, Pseudomonas aeruginosa, Escherichia coli, Staphylococcus aureus, Staphylococcus epidermidis, Bacillus subtilis, Bacillus thuringiensis, Bacillus subtilis, Salmonella typhi, Micrococcus luteus, Candida albicans, Klebsiella pneumoniae, Enterobacter cloacae and Streptococcus pyogenes [14,15,16,21,42,43,44]. Rahimullah et al. examined ethanol, chloroform, aqueous and hexane extracts from C. intybus seeds againts Escherichia coli and Staphylococcus aureus. All extracts showed activity against both organisms but aqueous extracts were more active and show a larger inhibition zone against S. aureus. Hexane, ethanol and chloroform extracts show significant zones of inhibition against both organisms. The authors of this study suggested that the medicinal plant has significant antibacterial activity and can utilize for the treatment and control of bacterial infections [15]. Methanolic and aqueous extracts of aerial parts of chicory were also investigated against Escherichia coli, Staphylococcus aureus, Staphylococcus epidermidis, Bacillus subtilis, Pseudomonas aeruginosa, Klebsiella pneumoniae, Erwinia carotovora, Proteus vulgaris, Enterobacter cloacae, Streptococcus pyogenes, and Candida albicans. The methanol extract exhibited broader antimicrobial spectrum than the aqueous extract compared with the standard antibiotics gentamicin and tobramycin. The water extract showed stronger activity against Staphylococcus epidermidis, Streptococcus pyogenes and Staphylococcus aureus, even more than that of the standard antibiotics [16]. Antibacterial activity of the water, ethanol and ethyl acetate extracts of C. intybus was investigated by Petrovic et al. All the tested extracts showed antibacterial activity, the ethyl acetate extract being the most active. A water extract inhibits Agrobacterium radiobacter sp. tumefaciens, Erwinia carotovora, Pseudomonas fluorescens and P. aeruginosa [14]. On the other hand, Shaikh et al. studied the antimicrobial properties of seed extracts using ethanol and ethyl acetate againts S. aureus, P. aeruginosa, E. coli and C. albicans. All the extracts showed significant antimicrobial activity against the tested microorganism, but S. aureus was found to be most sensitive and had the widest zone of inhibition. Aqueous extract showed maximum activity against S. aureus while did not show any significant activity against C. albicans. Ethyl acetate extract activity against P. aeruginosa and S. aureus was found to be significant [44].

### 4.3. Anti-Protozoal and Antiparasitic Properties

*C. intybus* is a plant with great potential against parasites, both in the context of livestock and humans [5]. This plant synthesizes several bioactive compounds with potential antiparasitic activity, but most studies have been devoted to the role of sesquiterpene lactones (SL). Woolsey et al. demonstrated the efficacy of chicory leaf and root extracts against *Cryptosporidium parvum*, with the lower SL extract (leaf) showing greater inhibition. Based on the results, the authors suggest that the antiparasitic activity does not appear to be related only to the SL content [45]. Marley et al. conducted a study on lambs that acquired parasites by natural transmission. The effects of chicory on total helminth parasites showed that this forage reduced the number of adult abomasal helminth parasites in lambs [46].

### 4.4. Hepatoprotective Properties

The hepatoprotective properties of extracts from various morphological parts of *C. intybus* have been confirmed in recent years by many authors [47,48,49,50,51]. One study, which used mice with liver previously damaged with acetaminophen and carbon tetrachloride, demonstrated, that application of a aqueous- and methanolic chicory seed extract to the animals lowered their mortality, as well as serum levels of alkaline phosphatase (AP), aspartate aminotransferase (AST) and alanine aminotransferase (ALT). Analogous results were obtained for alcoholic extracts of seeds and aqueous extracts of root and root callus–biochemical parameters, as e.g., elevated bilirubin levels, of treated animal health improved, and histological procedures confirmed significant alleviation of liver damage [1]. A number of liver damage markers, including low levels of superoxide dismutase, glutathione peroxidase, catalase and high levels of liver enzymes, were alleviated with a chicory-containing diet. Asadi et al. induced oxidative stress in rat liver with methotrexate [52]. This resulted in dramatic reduction in the levels of aspartate aminotransferase (AST), alanine aminotransferase (ALT), alkaline phosphatase (ALP), glutathione (GSH), catalase (CAT), superoxide dismutase (SOD) and glutathione peroxidase (GPx) and a significant increase in the levels of total bilirubin (TB) and malondialdehyde (MDA). The groups pretreated with C. intybus extratcs showed a significant increase in the levels of AST, ALT, ALP, GSH, CAT, SOD, and GPx and a significant decrease in the levels of TB and MDA. The extract of C. intybus seeds showed similar protective properties in a study, whereby rats were exposed to 4-*tert*-octophenol. In contrast, Ghaffari et al. studied the effects of turmeric and chicory seed supplementation (in a 1:3 ratio) on obesity markers and lipid profile in non-alcoholic fatty liver disease (NAFLD) in 92 patients. Turmeric supplementation alone and plus chicory seed led to significant reductions in serum levels of TG/HDL-C and LDL-C/HDL-C. In conclusion, turmeric and chicory seed supplementation can be significantly useful in management of NAFLD risk factors [53]. Most studies indicated that the positive effect of the extracts was related to their antioxidant activity [31,51,52].

### 4.5. Antidiabetes Properties

A study utilizing rats with streptozotocin-induced diabetes, found reduced serum levels of glucose, cholesterol and triglycerides upon application of whole-plant ethanolic extract at a dose of 125 mg/kg body mass. It was also determined that the chicory extract brought about a decrease in glucose-6-phosphatase levels, as compared with the control group. Similar hypoglycemic effects were observed using the aqueous seed extract, applied during either early or late phase of diabetes; it was observed that the extract prevents loss of body mass in the treated animals [1]. Pushparaj et al. investigated the effects of alcoholic extracts of whole chicory plants on rats with streptozotocin-induced diabetes (STZ, 50 mg/kg). Feeding chicory extract (125 mg/kg body weight) once daily for 14 days reduced serum glucose by 20%, triglycerides by 91%, and total cholesterol by 16%. The absence of changes in serum insulin levels ruled out the possibility that chicory induces insulin secretion by pancreatic β-cells. In addition, hepatic glucose-6-phosphatase (Glc-6-Pase) activity was significantly reduced. Decreased hepatic Glc-6-Pase activity may reduce hepatic glucose production, which in turn results in lower blood glucose levels in rats taking chicory extracts [31]. The aim of the study of Sharma et al. was to investigate the effects of *C. intybus* on lipid peroxidation activities of both enzymatic and non-enzymatic antioxidants, inflammatory mediators, myocardial enzymes and the histopathology of cardiac tissues in experimental diabetic cardiomyopathy (DCM). DCM was induced by single intraperitoneal injection of streptozotocin (40 mg/kg) combined with high energy intake in rats. Seed extract of *C. intybus* (250 and 500 mg/kg) was administered orally once a day for 3 weeks. Seed extract of *C. intybus* confirmed a significant potency towards restoring the blood glucose, an elevation of the levels of aspartate aminotransferase, lactate dehydrogenase, superoxide dismutase, thiobarbituric acid reactive substances, blood glutathione, TNF-α and IL-6 and a reduction in the levels of catalase was observed following the streptozotocin treatment. The extensive necrotic changes of cardiac tissue by streptozotocin was minimized to normal morphology upon *C. intybus* extract administration. The study demonstrates the cardioprotective effect of *C. intybus* extract via inhibition of oxidative stress and pro-inflammatory cytokines [54]. The caffeic and chlorogenic acids, found throughout the chicory plant, have been described as able antidiabetics, due to their effect on glucose reuptake in muscles. Both compounds are also potent inductors of insulin secretion. Another formerly mentioned compound of analogous properties is the cichoric acid, which also displays insulin-sensitizing and dose-dependent glucose tolerance-induction properties. Other data suggests that the whole-plant methanolic extract positively influences glucose transport, while not inducing adipogenesis at the same time [55]. Chadra et al. in a rat study showed that administration of an aqueous chicory seed extract significantly reduced serum glucose and triglyceride levels [56]. A later study by this author on 150 type 2 diabetes mellitus patients confirmed that ingestion of *C. intybus* water seed has significantly decreased inflammation, oxidative stress, and hypertriglyceridemia [33].

### 4.6. Effect on the Immune System

*C. intybus* is a medicinal plant commonly used in traditional medicine for its benefits in immune-madiated disorders. There are several evidences showing that *C. intybus* can modulate immune responses. Karimi et al. [57] studied the effect of ethanolic extract of chicory root on the immune system by targeting mouse dendritic cells. *C. intybus* at higher concentrations inhibited proliferation of allogenic T cells and in lower concentrations changed the level of cytokines such that IL-4 decreased and IFN-γ increased.

### 4.7. Analgesic Properties

The analgesic properties of three particular active substances of chicory—lactucin, lactucopicrin and 11β,13-dihydrolactucin—were proven in mice subjected to the hot plate (proving lactucopicrin the most potent) and the tail-flick test. It is noteworthy that a 30 mg/kg dose of any of these compounds used in the tail-flick test produced an effect comparable to that of a 60 mg/kg dose of ibuprofen. Furthermore, lactucin and lactucopicrin provided some extent of tranquilization as observed by reduction in spontaneous motor activity of the animals [1].

### 4.8. Anti-Cancer Properties

In recent years, cytotoxicity studies of *C. intybus* extracts have shown its antitumor potential [58,59]. The studies also identified metabolite constituents including guaianolides, 6-methoxyflavone, eudesmanolides, germacranolides, polyacetylene, sterol, anthocyanin, delphinidin, 3,4-dihydroxyphenethyl and other novel compounds. Many of these phytometabolites have shown positive cytotoxic activities in vitro, and antitumor action in vivo and in clinical trials, demonstrating the potential of *C. intybus* metabolites as antitumor drugs. Structural activity relationship studies have further confirmed these bioactivities [58]. *C. intybus* extracts have a cytotoxic effect among others on breast cancer (MCF-7), amelanotic melanoma (C32), prostate cancer (LNCaP), renal adenocarcinoma (ACHN), leukemia cells [59]. A study using Ehrlich ascites carcinoma mice proved a significant reduction in tumor progression upon application of raw ethanolic extract of the root. As high as 70% increase in average lifespan was observed upon intraperitoneal administration of 700 mg/kg body mass per day In turn, the aqueous-ethanolic macerate of the leaves suppressed proliferation of the C32 amelanotic melanoma cells [1].

### 4.9. Hypotensive Effect

According to Sedighi et al. ethanol leaves extract of *C. intybus* plays a protective role against hypertension. In this study, 32 male Wistar rats were divided into four groups of eight each. Animals in the control group were administered with normal saline and in the C. intybus groups with extract at 25, 50, and 100 mg/kg for two weeks. Median (MAP), systolic (SAP) and diastolic arterial pressure (DAP) significantly decreased in the 50 mg/kg extract-treated group compared to the control and 200 mg/kg extract treated groups [60].

### 4.10. Anti-Inflammatory Properties

Chicory is used externally in compresses useful for treatment of skin disorders, incl. dermatitis, inflammatory diseases of mucous membranes, ulcers, wounds and trauma. Flower infusion is excellent for treatment of inflammation-irritated skin and eyes, thanks to its antiseptic, anti-inflammatory, moisturizing and nutritious properties [61]. It also aids treatment of eczema and poorly healing wounds, improves periocular circulation, alleviates periorbital puffiness and symptoms of eye tiredness; it’s also excellent for dry skin care [61]. The medicinal materials of *C. intybus* are usually prepared as mixes with other herbs. Rizvi et al. evaluated the anti-inflammatory effects of root extracts by study rats. Chicory roots demonstrated significant dose-dependent decrease in paw edema in carrageenan-induced paw edema method. Chicory roots diminished the serum TNF-α, IL-6, and IL-1 levels. They also significantly attenuated the malonylaldehyde levels and increased the activities of CAT and GPx in paw tissue [62]. A placebo-controlled, double-blind, dose-escalating trial, was conducted to determine the safety and tolerability of a proprietary bioactive extract of chicory root in patients with osteoarthritis (OA). The results of the pilot study suggested that a proprietary bioactive extract of chicory root has a potential role in the management of OA. Only one patient treated with the highest dose of chicory discontinued treatment due to an adverse effects [63].

### 4.11. Anti-Neurotoxic Properties

*Cichorium intybus* contains glycosides and triterpenoids, which inhibit glutamatergic transmission and enhance GABAergic transmission. The present study was aimed at studying the effect of chicory extract on the pyridoxine-induced peripheral neuropathy with a particular focus on glutamatergic and GABAergic systems. In experimental study, a high dose of pyridoxine (800 mg/kg, i.p.) was injected for 14 days to induce neuropathy in male rats. Results showed beneficial effects of chicory extract on pyridoxine-induced peripheral neuropathy. Modulating of the GABAergic system mediated by TNF-α may be involved in the of chicory extract [64].The most important actions of chicory extracts are presented in the Table 3. 

## 5. Culinary Applications of the Common Cichory 

Roots, leaves and flowers are the most popular chicory parts for culinary and industrial applications [2,11,79,80]. The usage of chicory root as coffee substitute dates back to the XVIth century and continued through XVIIIth century, the discovery being attributed to the royal gardener Timme of Thuringen. Inulin, a polysaccharide present in roots, is converted during roasting into fructose and caramel, hence contributing to the dried roots’ dark hue and a pleasant taste with a bitter tinge, hence making it handy as a beer coloring. It is also useful for the preparation of syrup or sugar, subsequently used in the confectionery or alcohol production [7,11]. However, young roots can be boiled as is the case with similar root vegetables and served with sour cream or herbal sauce. Alternatively, they may be butter-fried, or cut into cubes and served to enhance the flavoring of various soups. Mature roots, after removal of the bitter core part, serve as a valuable addition to soups and meat dishes. Young leaves of the wild chicory variety may be prepared for salads, alone or with other leafy plants. Alternatively, they may be fried like spinach, and served with meat. Beforehand, they are often subject to bleaching to eliminate the bitterness, which is accomplished by burying the plant in soil at the beginning of spring, or by storing it in a dark cellar. Another, more facile, method to achieve this is to soak the leaves in boiling hot water before serving. Young leaves can also be ground, mixed with salt and stored for several months, for use in soups and sauces. They can also be boiled and served with tomato sauce, or added as spice to ham or cheese casseroles [2]. Chicory roots are also used to prepare drinks. The most popular are coffee surrogate. An interesting example is koumiss—a drink prepared traditionally by nomadic populations of Central Asia through fermenting mare milk. Nowadays, scientists used chicory root in order to reinvent this drink in a functional product with a positive impact on a cardiovascular and digestive level [81]. Another very interesting example is a functional drink obtained mainly from burdock root with added ginger juice and 10% chicory root. Such a functional drink has a unique organoleptic profile and diabetic patients can consume it as an aid to their therapy [82]. Thanks to their vivid color, fresh flowers are a perfect addition to salads, fish or as decoration for meat dishes [80]. Anthodia can be preserved in vinegar, and the buds can be marinated and served quite as the capparis are. Several other varieties of the chicory are cultivated. Apart from the *Cichorium intybus* L., there is also the so-called “salad chicory“ (*Cichorium endivia*), or “root chicory” (*Cichorium intybus* var. *radicorum* and *Cichorium intybus* var. *sativum*). The last two are a common material for coffee substitutes, while leaves of the *Cichorium intybus* var. *foliosum* are fit for salads. Chicory is most commonly served in salads, since its bitterness is well complemented by other popular salad components, e.g., nuts, varieties of blue cheese, apples or balsamic vinegar. It is also excellent served hot, e.g., under Béchamel sauce, although the British are known to prepare a soup of it. Insurmountable are the ever-coming propositions for chicory-containing meals, such as: chicory root salad with vinaigrette sauce, asparagus style-served roots, marinated buds, crepe cake, spinach-chicory or tomato-chicory mixes, chicory rolls with ham and parmesan cheese, braised chicory with blackberries, young leaf salad with vinaigrette sauce, salad of chicory root, pickled flower buds.

## 6. Allergies to the Common Cichory

A rare allergy to the chicory plant has been documented in ~20 cases over the last 100 years. Most of these cases occurred in adults who were in contact with chicory due to their occupation, and only a single case involved a child reacting to inulin. Depending on the individual, allergic symptoms can be systemic and/or local, ranging from rhinoconjunctivitis, to asthma and anaphylactic reactions, to contact dermatitis. As individual as the allergic reactions are, the chicory preparations and routes of exposure are also unique and varied. There are only two reported cases where fresh chicory roots induced an allergic reaction topically. The majority of reactions occurred in response to leaves (raw and cooked) after skin contact or inhalation. Sometimes, reactions were also caused by the inhalation of dried chicory roots and inulin, consumption of inulin-containing products, and once by intravenous inulin administration during a standard renal function test. It is not yet clear exactly how allergic reactions to chicory are triggered. Proteins from chicory or newly formed inulin-protein compounds (arising during production), as well as sesquiterpene lactones could be potential allergens. Sensitization might arise from repeated exposures or from cross-sensitization with birch pollen or lettuce. Due to all this ambiguity, the general advice is that people with allergies or occupational exposure to Asteraceae family members, people with birch-pollen allergies, and people with atopic dermatitis should be cautious when coming into contact or consuming chicory- and inulin-containing foods [2,83].

## 7. Conclusions

In conclusion, *Cichorium intybus* L. is a common plant with great potential. It certainly does deserve a wider use in medical prophylaxis and phytotherapy. Individual parts, e.g., leaves or flowers, both in fresh and dried form, can be a valuable addition to daily diet. The multipurpose effects of *C. intybus* extracts may be a promising alternative source for the pharmaceutical industry. It is interesting to note that chicory was among the plants with potential against SARS-CoV-2. However, further studies, including in vitro and in vivo studies, are needed to confirm this antiviral property of chicory.

## Figures and Tables

**Table 1:** Chemical composition of *Cichorium intybus*.

Part Plant	Chemical Compounds	References
Leaves	fatty acids: C14:0; C15:0; C16:0; C18:0; C18:1n-9c; C18:2n-6c; C18:3n-3; C20:0; C22:0; C24:0	[18]
	pigments: lutein, violaxanthin antheraxanthin, neoxanthin, chlorophyll a, chlorophyll b, pheophytin a, pheophytin b, β-carotene	
	polyphenols, flavonoids, chlorogenic acid, caffeic acid, chicoric acid, quercetin glucuronide, gallic acid	
	tannins, saponins	[11,19]
	minerals: Ca, Mg, Na, Cu, Zn, Mn, Se, N, P, K, S, B, Fe	
	vitamins: A, E, K, C, B1, B2, B3, B5, B6, B9	[20]
Roots	polyphenols, flavonoids, caffeoylquinic acid,	[21]
	minerals: Ca, Mg, Na, Cu, Zn, Mn, Fe, K,	[11,22]
	phenolic compound: protocatechuic acid, chlorogenic acid, hydroxybenzoic acid, isovanillic acid, coumaric acid, protocatechuic acid, chlorogenic acid, caffeic acid, coumaric acid, *p*-coumaric acid	
	fatty acids: C_14_H_28_O_2_, C_16_H_30_O_2_, C_16_H_32_O_2_C_20_H_34_O_2_, C_20_H_40_O_2_	[23]
	steroids, terpenoids,	
	vitamin C	[22]
	tannins	
Seeds	amino acids: arginine, histidine, isoleucine, leucine, lysine, methionine, cysteine, phenylalanine, tyrosine, threonine, valine, serine, glutamic acid, glycine, alanine, aspartic acid, proline	[22,24]
	fatty acids: C14:0, C16:0, C16:1, C18:0 t11-C18:1, C18:1n-9, C18:2n-6, C18:3n-3, C20:0, C20:1, C20:2, C22:0, C24:0	
	minerals: P, K, Ca, Mg, Na, Fe, Cu, Zn, Mn, Mo, Se, Cd,	
	steroids, terpenoids,	[23]
	vitamin C	[22]
	tannins	
Flowers	fatty acids: fatty acids: C_14_H_28_O_2_, C_15_H_30_O_2_, C_16_H_32_O, C_17_H_34_O_2_, C_18_H_32_O_2_, C_18_H_36_O_2_, C_19_H_38_O_2_, C_18_H_30_O_2_, C_20_H_40_O_2_, C_20_H_40_	[23]
	steroids, terpenoids,	
Aerial parts	phenolic compounds: caftaric acid, chlorogenic acid, cichoric acid, isoquercitrin, rutin, quercitrin, luteolin, apigenin	[25]
	minerals: Fe, Cu, Zn, Mn	[22]
	vitamin C	
	tannins	

**Table 2:** The chemical compounds isolated from *Cichorium intybus*.

Part Plant	Essential Oil	References
Aerial parts	carvacrol (50.1%); thymol (13.3%); cinnamic aldehyde (12.4%); camphor (4.4%); carvone (4.1%); linalool (3.9%); α-terpineol (2.1%); octane (8–25.6%); octen-3-ol-1 (0.3%); 2-pentylfuran (up to 2.6%); (2*E*,4*E*)-heptadienal (up to 2.6%); 1,8-cineole (up to 1.0%); phenylacetaldehyde (1–4.5%); *n*-nonanal (2.1–6.5%); camphor (1.4%); (2*E*,6*Z*)-nonadienal (0.6%); (2*E*)-nonen-1-al (0.9%); *n*-decanal (0.8–1.7%); (2*E*,4*E*)-nonadienal (up to 0.4%); *n*-decanol (up to 0.9%); (2*E*,4*Z*)-decadienal (0.5–1.3%); (2*E*,4*E*)-decadienal (1.5–1.9%); geranyl acetone (0.7–3.2%); β-ionone (1.9–3.0%); (2*E*)-tridecanol (6.3%); pentenyl salicilate (0.9%); *n*-hexadecane (0.9–5.9%); tetradecanal (1.0–2.8%); tetradecanol (0.8%); 2-pentadecanone (4.2–14.9%); (*E*)-2-hexylcinnamaldehyde (0.4%); octadecane (0.5%); *n*-nonadecane (5.1–46.9%); (5*E*,9*E*)-farnesyl acetone (0.6–2.3%); *n*-eicosane (0.9–2.9%); *n*-octadecanol (0.3–1.0%); *n*-heicosane (2.5–8.0%)	[26,27]
Roots	Kaempferol; octane (34,3–69.8%), octen-3-ol-1; 2-pentylfuran; *n*-nonanal (up to 1.2%); *n*-tridecane (0.3–0.4%); (2*E*,4*E*)-decadienal (2.2–3.4%); (2*E*,4*Z*)-decadienal (0.8–0.9%); (2*E*,4*E*)-heptadienal (up to 1.0%); β-elemene (0.3–0.6%); (*E*)-caryophyllene (up to 0.4%); β-ylangene (0.3–0.7%); (*E*)-β-farnesene (0.4–2.2%); geranyl acetone (up to 0.6%); allo-aromadendrene (up to 3.9%); dehydro-aromadendrene (0.6%); β-ionone (0.5%); pentadecane (1.8%); *trans*-β-guaiene (0.5–0.7%); (2*E*)-undecenol acetate (1.3–1.9%); sesquicineole (up to 0.8%); (2*E*)-tridecanol (0.5–2.6%); pentenyl salicilate (4.8–22.7%); *n*-hexadecane (1.7–18.1%); tetradecanal (1.1–2.7%); 2-pentadecanone (0.4–1.3%); *n*-nonadecane (0.3–3.9%); *n*-eicosane (2.1–5.1%); *n*-heicosane (0.4–0.5%)	[27,28]

**Table 3:** Chicory extracts and their activity in different disorders.

	Extract/Used Part of Plant	Therapeutic Action	Research Model	References
1.	Water, ethanol/roots	Hepatoprotective	Albino Wistar rats	[65]
2.	Hydroalcoholic			[52]
3.	Water/leaves			[66]
4.	Combined extract of jujube, chicory and silymarin			[50]
5.	Methanol/leaves			[49]
	Methanol/roots			
6.	Alcohol/seeds			[47]
7.	Hydroalcoholic/fruits		Sprague Dawley rats	[67]
8.	Ethanol/aerial part	Hepatoprotective and antioxidant	Female Albino Wistar rats	[68]
9.	Water/seeds	Antidiabetic, antioxidant	Wistar rats	[56]
10.	Ethanol/whole plant	Antidiabetic	Sprague-Dawley rats	[31]
11.	Hexane/whole plant	Antidiabetic, antioxidant	Diabetic mice	[69]
12.	Water/seeds	Anti-inflammatory, antioxidant, antidiabetic	150 patients	[33]
13.	Methanol/roots	Anticancer	Breast Cancer (SKBR3) cell line	[70]
14.	Ethanol/aerial part		Colon cancer (HCT 116), liver cancer (HEPG2) cell lines	[68]
15.	Water/seeds	Cardioprotective	Albino Wistar rats	[54]
16.	Commercial roots extract	Improve skin barrier function	Fifty women	[71]
17.	Chicory inulin—commercial product (Fibrulose F97; Cosucra).	Decreased serum uric acid, triglyceride, and abdominal fat deposition in a quail model of hyperuricemia by altering the acetyl-CoA carboxylase protein expression and fatty acid synthase and xanthine oxidase activities.	French quails	[72]
18.	Hydroalcoholic/roots	Protective in pancreatitis	Mice	[73]
19.	Ethyl acetate, hydroalcoholic, hexane/roots	Antiinflammatory, prebiotic	Human colon carcinoma (HT29) cell line	[74]
20.	Water/seeds	Antiinflammatory, modulating expression of cytokines	Wistar albino rats	[51]
21.	Root extract—commercial product	Antiinflammatory	40 patients	[63]
22.	Water/seeds	Hepatic steatosis caused by early and late stage diabetes	HepG2 cell line	[75]
23.	Hydroalcoholic/roots	Antiproliferative	Renal adenocarcinoma (ACHN), amelanotic melanoma (C32), breast cancer (MCF-7), prostate cancer (LNCaP) cell lines	[76]
	Hydroalcoholic/leaves			
24.	Water, methanol/roots	Gastroprotective	Spraque-Dawley rats	[77]
25.	Seeds + turmeric	Decrease NAFLD risk factors	92 patients	[53]
26.	Hydroalcoholic/roots	Antineurotoxic, neuroprotective	Albino Wistar rats	[64]
27.	Ethanol/leaves	Improve reproductive parameters	Adult male Wistar rats	[78]

## Data Availability

Not applicable.
